# ECG Smart Monitoring versus Implantable Loop Recorders for Atrial Fibrillation Detection after Cryptogenic Stroke—An Overview for Decision Making

**DOI:** 10.3390/jcdd10070306

**Published:** 2023-07-18

**Authors:** Thomas Pezawas

**Affiliations:** Department of Medicine II, Division of Cardiology, Medical University of Vienna, Waehringer Guertel 18-20, 1090 Vienna, Austria; thomas.pezawas@meduniwien.ac.at; Tel.: +43-1-40400/46140

**Keywords:** ECG monitoring, loop recorder, stroke, atrial fibrillation

## Abstract

Up to 20% of patients with ischemic stroke or transient ischemic attack have a prior history of known atrial fibrillation (AF). Additionally, unknown AF can be detected by different monitoring strategies in up to 23% of patients with cryptogenic or non-cardioembolic stroke. However, most studies had substantial gaps in monitoring time, especially early after the index event. Following this, AF rates would be higher if patients underwent continuous monitoring early after stroke, avoiding any gaps in monitoring. The few existing randomized studies focused on patients with cryptogenic stroke but did not focus otherwise specifically on prevention strategies in patients at high risk for AF (patients at higher age or with high CHA2DS2-VASC scores). Besides invasive implantable loop recorders (ILRs), external loop recorders (ELRs) and mobile cardiac outpatient telemetry (MCOT) are non-invasive tools that are commonly used for long-term ECG monitoring in cryptogenic-stroke patients in the ambulatory setting. The role of MCOT and hand-held devices within ECG smart monitoring in the detection of AF for the prevention of and after cryptogenic stroke is currently unclear. This intense review provides an overview of current evidence, techniques, and gaps in knowledge and aims to advise which patients benefit most from the current available devices.

## 1. Introduction

Stroke is one of the leading causes of death and disability worldwide. In ischemic stroke, comorbidities can suggest an embolic cause, but frequently a specific source cannot be identified. Following this, the term “embolic stroke of undetermined source” (ESUS) designates patients with non-lacunar ischemic stroke where embolism could be the likely stroke mechanism [1]. Despite advancements in medical technology, the cause of ischemic stroke remains undetermined in approximately one third of cases [2]. This is also termed “cryptogenic stroke” (CS). Risk of stroke is increased five-fold in atrial fibrillation (AF) [3] independently of whether AF is paroxysmal or persistent [4]. Up to 20% of patients with ischemic stroke have a previous history of AF, but incidence rates increase steeply with age [5]. In stroke patients >80 years of age the incidence of AF-related stroke has increased three-fold during the last 35 years, and another three-fold increase is expected by 2050 [5]. Detection of AF is of central importance after stroke or transient ischemic attack (TIA), as risk of recurrent stroke can be effectively reduced by oral anticoagulation with warfarin [6] and novel oral anticoagulants [7]. This strategy has a huge impact on secondary prevention [8]. Prolonged electrocardiogram (ECG) monitoring is appropriate in patients with CS and transient ischemic attack (TIA) who have a negative baseline diagnostic workup. This workup usually includes inpatient telemetry and at least 24 h outpatient Holter monitoring [8]. The strategy of long-term monitoring is used to substantially increase the probability of AF detection in patients with CS. When AF is detected in time, early initiation of anticoagulation therapy can have a huge impact on recurrent-stroke prevention in these patients [9]. Long-term electrocardiogram (ECG) monitoring tools are commonly used for paroxysmal AF detection, and proof of their accuracy and the reliability of detection algorithms should be of eminent interest. Current methods of ECG monitoring include invasive and non-invasive methods [10]. External loop recorders (ELRs) and mobile cardiac outpatient telemetry (MCOT) are ambulatory non-invasive diagnostic tools that are commonly used for long-term ECG monitoring in CS patients [11]. The accuracy of mobile cardiac outpatient telemetry (MCOT) in the detection of AF after cryptogenic stroke is currently unclear. Promising non-invasive tools such as novel mobile-health (mHealth) options for long-term ECG monitoring are arising, but their reliability and accuracy in the setting of CS have to be proven. Devices with the highest proven yield in detecting AF are implantable cardiac monitors (ICMs) and implantable loop recorders (ILRs). This is true especially in patients with CS, since ICMs prolong substantially the duration of cardiac monitoring (≥5 years) and use remote monitoring [12]. Compared to conventional follow-up with scheduled ECG monitoring, both ILRs and MCOT have by far the highest rates of AF detection [8,11,13]. Due to the relatively costly technology, the accuracy of ICMs should be improved for further implementation of this technology in ischemic-stroke patients. The use of artificial intelligence (AI) or other arising techniques may improve the detection algorithms of ICMs suffering from false-positive detection rates [14].

In accordance with the 2021 American Heart Association/American Stroke Association guidelines for the management of acute ischemic stroke, the best duration of extended ECG monitoring after acute ischemic stroke is still uncertain [15]. Existing systematic-review papers have their main focus on the assessment of the efficacy of ILRs in different settings [10,16], with a relative lack of literature investigating the use of MCOT after CS or comparing face to face the efficacy of ILRs with MCOT. Furthermore, the need for an invasive procedure when implanting ILRs and the resulting extended follow-up is not accepted by some patients [17]. Although health-technology assessments have proven their efficacy, ILRs are still expensive and are considered cost effective if continuously used over a three-year period [18]. Recently, promising data regarding the efficacy of MCOT have been shown in some individual studies. These data suggest that MCOT may replace or could be used in combination with ILRs [10,13].

This systematic review tries to compare the differences between ILRs and MCOTs in the detection of AF following CS. Secondary aims are to determine AF-detection rates regarding different ECG techniques, to identify factors influencing the rate of AF detection between different modalities, and last but not least, to identify patients who will benefit most from ILRs and MCOT.

## 2. Technologies

**Figure 1:**
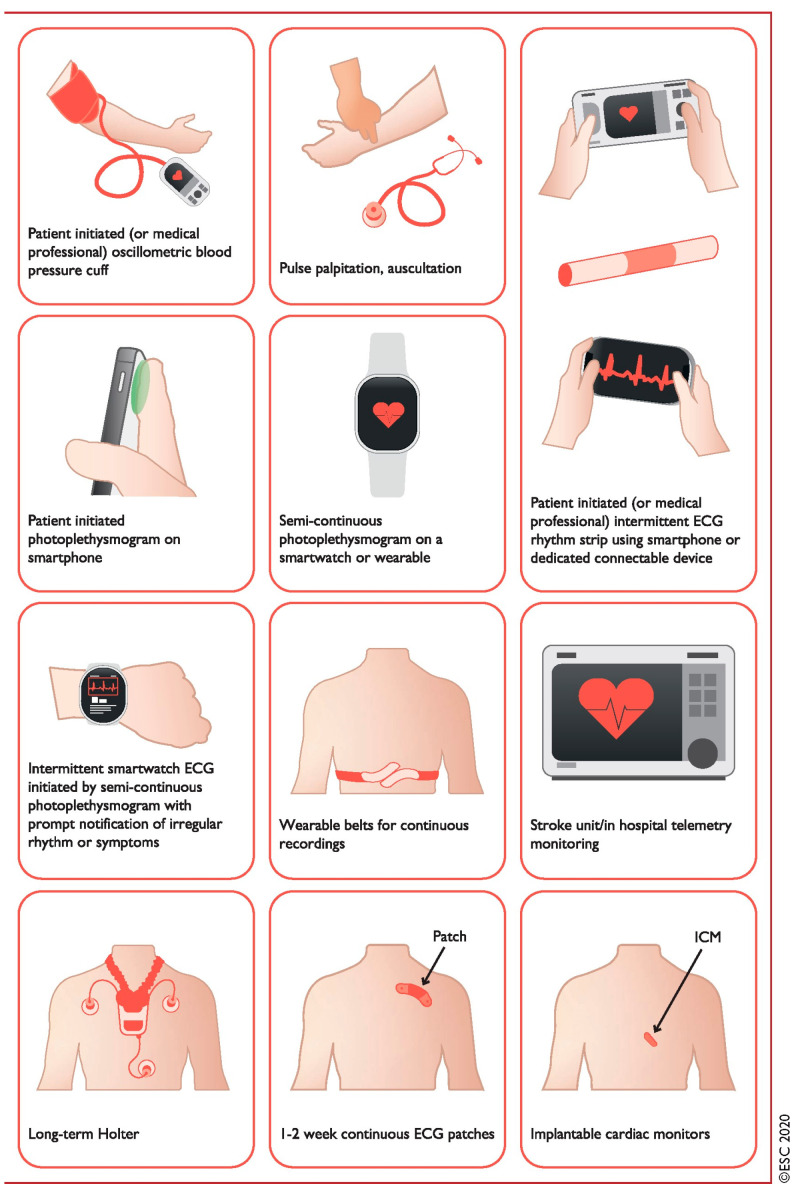
Systems used for AF screening. Pulse palpation, automated BP monitors, single-lead ECG devices, PPG devices, and other sensors (using seismocardiography, accelerometers, gyroscopes, etc.) used in applications for smartphones, wristbands, and watches. Intermittent smartwatch detection of AF is possible through PPG or ECG recordings. Smartwatches and other wearables can passively measure pulse rate from the wrist using an optical sensor for PPG and alerting the consumer of a pulse irregularity (based on a specific algorithm for AF detection analyzing pulse irregularity and variability). AF = atrial fibrillation; BP = blood pressure; ECG = electrocardiogram; PPG = photoplethysmography. Reprinted with permission from Ref. [19]. 2021, Gerhard Hindricks.

An overview of current technologies is outlined in Figure 1 and Table 1.

### 2.1. Handheld Devices

Handheld devices are electronic devices that are small enough to be held in the palm of the user. These devices are portable and designed to be activated by touching and are capable of detecting, analyzing, and transmitting bio-signals. Currently, three technologies are available to detect and to monitor AF: photoplethysmography (PPG)-, electrocardiography (ECG)-, and mechanocardiography (MCG)-based devices [20]. These include mainly FibriCheck [21], CardiioRhythm [20], Preventicus [22], PULSE-SMART [23], and Kardia mobile by AliveCor [24], and many others are currently being introduced on the market.

### 2.2. Wearable Devices

Wearable devices are electronic devices that are worn on the body and are designed to be lightweight so they can be integrated into clothing or worn like an accessory. Wearables are characterized by their ability to collect data about the user, such as heart rate, ECG, activity, and sleep patterns. They are particularly popular among people who want to monitor their physical status with a wide range of PPG-based devices. Wearables can be designed as rings, armbands, wristwatches/bands, armbands, rings, and earlobe sensors. ECG-based devices can be designed as patches, chest belts, and wireless recorders. Finally, pulse-variability-based devices are devices such as sphygmomanometers. Due to the huge amount and continuous development and presentation of new devices, only the method is listed with names of current known products:

*Photoplethysmography-based wearables:* These include Apple Watch, Samsung Galaxy Watch, Fitbit, Empatica E4, and CardiacSense (all cleared by CE and the FDA). Storage of PPG recordings and access for patients is managed by secure cloud solutions. Currently, the following devices provide these solutions: Apple Watch, Samsung Galaxy Watch, Fitbit, CardiacSense, Samsung Simband, Empatica E4, Gear Fit 2, Wavelet Health, Amazfit, Honor Band 4, Huawei Watch GT, and Honor Watch.

*Electrocardiography-based wearables*: ZioXT (FDA approved), RhythmPad, Firstbeat Bodyguard 2 (CE approved), and Medi-Trace 200 (validated in clinical studies, FDA and CE approval). Usually, patches use a single-lead or a three-lead ECG recording that is placed on the patients’ chest. In contrast, RhythmPad consists of three sensors placed around both arms and the right leg to record a six-lead ECG. The monitoring duration varies between 10 s and two weeks. TWH (Nuubo) is a textile Holter monitor that can be worn for 90 days.

*Pulse-variability-based wearables:* These include Microlife BP and OMRON (validated in clinical studies, FDA and CE approval). Storage of PPG recordings and access for patients is managed by secure cloud solutions.

### 2.3. Implantable Loop Recorders

ILRs are small devices that are implanted under the skin in the chest area to continuously record ECGs. They can be triggered manually by the patient by an external handheld device or a smartphone. Due to the continuous-monitoring capability, activation is carried out automatically by software routines. ILRs have a broad spectrum of other established indications for use [25]. Wireless communication is possible between the clinician programmer, the ILR, the smartphone, or other computing devices. Via different applications, patients receive information about the heart rhythm, possible rhythm disturbances, and when they should get in contact with the treating doctor. Currently, ILRs are available from four main manufactures: Reveal (Medtronic), BioMonitor (Biotronik), Lux (Boston Scientific), and Confirm (Abbott).

### 2.4. Mobile Platforms and Support Systems

There is widespread use of mobile devices (smartphones, watches, and tablets), and users spend time on mobile applications most of all. In 2017, 318,000 mobile applications were available worldwide, including more than 500 dedicated to AF management [26].

## 3. Discussion

### 3.1. Overview of Current Evidence

Monitoring methods have been tested in several observational studies [27,28,29], with average detection rates of atrial fibrillation of about 10% and with a higher yield in studies screening selected patients (mostly cryptogenic stroke).

Four randomized, controlled studies of cardiac monitoring after IS/TIA have been published: two smaller pilot studies (one analyzing MCOT [30] and the other a 7-day external loop recording [31]) and two randomized controlled trials analyzing 30 days of external loop recording [13] and implantable loop recording [8]. Taken together, the results of the two large randomized controlled trials (EMBRACE and CRYSTAL AF) suggest that approximately 10 patients need to be screened with prolonged monitoring to establish one new patient diagnosis of atrial fibrillation. Based on these published data and expert opinion, guidelines [32] suggest that prolonged rhythm monitoring (≈30 days) for AF is reasonable within 6 months of the index event. However, even though, according to published guidelines, additional ECG monitoring by long-term non-invasive ECG monitors or implanted loop recorders should be considered in stroke patients to document silent atrial fibrillation, in clinical practice most patients still do not receive any extended ambulatory external or implantable ECG monitoring. A reason for that might be the restricted availability of external event recorders, that it is not well established, or due to possible inter-disciplinary cooperation or ILR-associated costs [33].

A shortcoming of previous studies is the relatively long delay between hospital discharge and the start of ambulatory ECG monitoring. In the randomized controlled trials EMBRACE and CRYSTAL AF, patients were randomized to ECG-monitoring techniques for 75.1 ± 38.6 days and 38.1 ± 27.6 days after the qualifying event, respectively.

Four sequential phases of AF screening were reported in a meta-analysis [28] of studies reporting the detection of post-stroke AF: Phase 1 consisted of admission ECG; phase 2 comprised serial ECG, continuous inpatient ECG monitoring, continuous inpatient cardiac telemetry, and in-hospital Holter ECG monitoring; phase 3 consisted of ambulatory Holter ECG monitoring; and phase 4 consisted of mobile cardiac outpatient telemetry, external loop recording, and an ILR. Overall, according to this meta-analysis, in 23.7% of patients with ischemic stroke AF could be detected by screening. The probability of detecting AF was 7.7% in phase 1, 4.2% in phase 2, 7.5% in phase 3, and 4.3% in phase 4.

Therefore, in about a third of patients, AF can be detected by the first ECG at admission and in about half of the patients by in-hospital ECG screening. However, in most of the studies included into phase 2, the duration of in-hospital ECG screening was short (i.e., <72 h). Only few previous studies [31,34,35,36,37] analyzed prolonged ECG monitoring (≥7 days) within 14 days of the cerebrovascular event by various methods (Holter ECG monitoring, external loop recorders) and yielded detection rates of 10–20% compared to standard monitoring (12-lead ECG and/or 24 h Holter ECG). In the randomized controlled pilot study by Higgins et al., 100 patients with ischemic stroke were randomized either to 7 days of noninvasive cardiac-event monitoring or to standard monitoring (12-lead ECG and 24 h Holter ECG monitoring) [31]. The external devices were applied within 7 days after the index event. After 90 days, sustained episodes of AF were detected significantly more often in patients undergoing prolonged cardiac-event monitoring (22% vs. 8%). Recently, two large randomized studies compared 7-day Holter monitoring [38] and 10-day Holter monitoring [39] early after IS. The first (MonDAFIS) did not find an effect of systematic ECG monitoring on the rate of oral anticoagulation use after 12 months. The second (FIND-AF Randomised), showed that enhanced and prolonged monitoring early in patients with acute stroke aged 60 years or older was better than standard care for the detection of AF (14% vs. 5% after 6 months).

So far, there has only been one small observational study [40] analyzing incidence rates of AF using ILRs early after IS. In the randomized controlled study CRYSTAL AF [8], the median time to randomization was 38.1 ± 27.6 days. In the randomized study STROKE-AF [41], which analyzed incidence rates of AF in stroke patients with known etiology by comparing continuous cardiac monitoring using an ILR with standard monitoring, patients were randomized within 10 days but not using a gapless approach. STROKE-AF [41] and the recent PER-DIEM [42] and MonDAFIS studies [38] showed that prolonged ECG monitoring by an ILR produced high AF-detection rates not only in patients with CS but also in patients with small-vessel or large-artery disease. However, STROKE-AF [41] had relatively short ILR monitoring times and included patients with small-vessel or large-artery disease only. In contrast, monitoring time was longer (11–21 h) in the MonDAFIS study [38] and additional 7-day monitoring was applied.

Very recently, the GEMS-AF study (Gapless Electrocardiogram-Monitoring in Stroke at High Risk of Atrial Fibrillation Study) [43] demonstrated for the first time in 110 patients that gapless ECG monitoring without any interruption in monitoring time is a feasible and effective approach to substantially increase detection rates of AF after an ischemic stroke. In GEMS-AF [43], the detection rate of AF within the first 30 days was 10.0%, which accounted for two thirds of all new AF diagnoses. The median duration of the detected episodes was 1.7 (0.2–4.7) hours. The detection rate of AF after 6 months was 15.5%. Relevantly, in GEMS-AF [43], ILR implantation occurred after thorough telemetric ECG monitoring on stroke units with a median duration of 70 h. During telemetry, AF was detected in another seven cases, which were not included in the final analysis.

### 3.2. AF Duration and Type of Stroke

It is still an open question which duration of AF is associated with an increased stroke risk. Whereas a 2 min cutoff is determined for AF-detection algorithms, within ILR devices a 30 s cut-off is generally implemented based on expert consensus. Most studies used a 30 s duration as a threshold [44]. This 30 s cutoff duration has to be seen in a historic context, since it was established from the definition of non-sustained ventricular tachycardia (VTs below 30 s duration). Accordingly, in clinical practice, most physicians use this threshold for initiation of anticoagulant treatment in patients with AF after stroke/TIA. However, data from the population-based OXVASC study questioned this approach [45]. In that study, patients after stoke/TIA underwent ambulatory ECG monitoring (Novacor-R test). Patients in whom AF was detected were stratified based on AF duration (<2 min/≥2 min). Of note, patients with AF duration <2 min were not anticoagulated. At the end of the study (mean follow up: 2.3 years), those with an AF duration of <2 min had a similar risk of recurrent stroke as patients without AF. It has to be stated that the 2 min threshold has additionally been used by manufacturers of ILRs for the minimum AF duration to detect AF as AF. Therefore, additional evidence will also have on impact on programming possibilities for ILRs.

Studies analyzing prolonged-monitoring methods, including the EMBRACE [13] and CRYSTAL AF studies [8], mostly focused on patients with CS. However, in a population-based study of patients with ischemic stroke or TIA, risk of newly detected AF as well as of AF-related cardioembolic events was similar in patients with CS and those with non-cardioembolic stroke (i.e., large-artery-disease and small-vessel-disease events combined) [46]. A randomized pilot study of noninvasive cardiac-event monitoring included unselected patients with non-cardioembolic stroke and reported high detection rates of AF (see above) [31]. Therefore, studies focusing exclusively on patients with CS (or ESUS) [47] might underestimate proportions of AF and demand of oral anticoagulation. Yet, the selection of patients at particularly high risk for AF and recurrent cardioembolic events after stroke or TIA is crucial. Clinical scores have been developed to assess risk stratification for cardioembolic events in patients with AF (i.e., CHADS2 score and CHA_2_DS_2_VASc score) and have been associated with total duration of AF (lower CHADS2 scores in patients with low AF burden). In addition, previous studies have also indicated the usefulness of the CHADS2 and CHA_2_DS_2_VASc scores for prediction of AF in patients after stroke [48,49]. In the study by Baturova et al., CHADS2 scores ≥ 4 and CHA_2_DS_2_VASc scores ≥ 5 predicted new-onset AF in patients after ischemic stroke during 10 years of follow-up [49]. Importantly, this study did not focus on cryptogenic stroke specifically but rather reported data on an unselected population of ischemic stroke.

### 3.3. Prevention Strategies

Relevantly, detection of atrial fibrillation cannot be equated with prevention of cerebrovascular and peripheral embolic events. None of the previous studies analyzing different strategies of ECG monitoring after stroke were powered to show an effect on the prevention of clinical events. A meta-analysis of an ILR after stroke including the CRYSTAL AF, STROKE AF, and PER DIEM studies did not detect a significant reduction in cerebrovascular events. However, despite including data of 1233 randomized patients, the number of recurring events was low (n = 94) and below the approximately 500 stroke events needed to adequately power a definitive evaluation of stroke reduction with an ILR [50,51]. However, in a meta-analysis of prolonged ECG monitoring after stroke/TIA not exclusively focusing on ILRs and also including observational studies, prolonged monitoring was associated with a lower risk (RR 0.58, 95% CI 0.41, 0.82) of recurrent stroke [52]. Previous studies suggested that covert brain infarction (CBI) could be used as a surrogate for assessing stroke therapies [50,53]. In the published PACIFIC stroke trial, the occurrence of CBI was three times higher than of clinical stroke events [54]. Patients with CBI are at increased risk of subsequent clinically manifest ischemic stroke [55]. In summary, these data show that diagnosis of CBI in patients after acute ischemic stroke is of value, in particular in patients with atrial fibrillation, and that CBI can be used as a surrogate for clinical manifest ischemic stroke in clinical studies. Other strategies, such as wide and systematic AF screening for prevention of stroke by event recording, failed, such as the 2021 published STROKESTOP study [56].

### 3.4. Techniques

Because atrial fibrillation is known to be one of the strongest risk factors for stroke, anticoagulation therapy has been shown to be highly effective in reducing AF-related strokes [57]. This proven benefit of anticoagulation has been demonstrated in clinical studies, where clinically relevant AF and asymptomatic AF detected by prolonged ECG monitoring have been investigated [58]. The association between stroke and AF is probably accelerated by an underlying atrial cardiopathy [59]. Undoubtably, AF itself increases stroke risk [60]. Other strong risk factors for stroke are cardiovascular comorbidities, which, on the one hand, elevate the burden of AF, a a higher burden of which increases the risk of stroke by itself. On the other hand, patients with a history of stroke have, by definition, a higher probability of a re-stroke than otherwise healthy patients with lone atrial fibrillation only [61]. Once a recent stroke has occurred, newly diagnosed AF is associated with an increased risk of a re-stroke [62]. In addition, higher age and vascular comorbidities increase the likelihood of AF detection via prolonged monitoring in these patients [63]. Importantly, in the setting of a re-stroke, greater disabilities can be seen compared to non-AF-related re-stroke [64]. Keeping this in mind, the increasing value of prolonged ECG monitoring is evident, compromising low-cost smartphone-compatible devices and ILRs that can provide five or more years of continuous ECG monitoring [65]. The setting of ILR ECG monitoring of three years or more was shown to have clear advantages [42]. The close relationship between the thrombogenic potential of atrial cardiopathy and AF itself, as well as newly diagnosed AF in ESUS patients, has therapeutic implications. We might not know this in detail until the full results of the ARTESiA (Apixaban for the Reduction of Thrombo-Embolism in Patients with Device-Detected Sub-Clinical Atrial Fibrillation) and NOAH (Non-Vitamin K Antagonist Oral Anticoagulants in Patients with Atrial High-Rate Episodes) trials are available. Meanwhile, a relatively harmless and potentially beneficial implantation with an ILR in this group of stroke patients seems to be the adequate strategy to protect patients from re-stroke.

### 3.5. Knowledge Gaps for Atrial-Fibrillation Detection after Cryptogenic Stroke

There may be a critical burden of AF above which anticoagulation is effective that varies depending on the overall risk profile. For example, an otherwise healthy young patient with AF does not require anticoagulation, but when an ESUS patient crosses a threshold to justify anticoagulation is unknown. Herein lies the controversy of what is considered clinically meaningful AF. What level of burden of AF increases stroke risk and what is the temporal relationship between AF and stroke? Therefore, scores may not accurately assess future stroke risk in patients with ESUS with a low burden of AF identified via intensive monitoring. Furthermore, anticoagulation has yet to be proven effective in patients with only low-burden AF detected by intensive rhythm monitoring.

Stroke-risk stratification using CHADS_2_ [61] and CHA_2_DS_2_-VASc [66] was investigated with AF-screening methods that are considered less sensitive. Following this, CHADS_2_ and CHA_2_DS_2_-VASc were determined to not be accurate enough for future stroke patients, especially ESUS patients who are known to have a relatively low burden of AF. Given the low burden of AF detected by intensive ECG monitoring, the efficacy of anticoagulation therapy needs further approval in this patient setting. To date, the critical burden of AF detected with ILRs to initiate anticoagulation therapy is unknown. Furthermore, the effectiveness of anticoagulation in low-AF-burden patients needs more confirmation from clinical trials. The controversies of the proven benefits of anticoagulation in high-risk patients and the unclear benefits of anticoagulation due to missing cutoff levels of detected AF or AF burden are evident [67].

However, how can we overcome these controversies? Important sources are pacemaker and defibrillator studies demonstrating that hours of AF are needed to significantly increase stroke risk [68,69]. These studies also point to the dilemma that short-term AF accidently found months after the index stroke may not be related to the stroke event. Moreover, we have to ask ourselves whether these short telemetric-recorded AF episodes justify life-long treatment with anticoagulants in ESUS patients. In turn, AF-related strokes are known to be more disabling and are associated with worse outcome [70]. For instance, in the CRYSTAL AF study [8], the mean baseline National Institutes of Health Stroke Scale score was relatively low (1.6 ILR versus 1.9 control). In NAVIGATE ESUS (New Approach Rivaroxaban Inhibition of Factor Xa in a Global Trial Versus ASA to Prevent Embolism in Embolic Stroke of Undetermined Source) [71] and RE-SPECT ESUS (Randomized, Double-Blind Evaluation in Secondary Stroke Prevention Comparing the Efficacy and Safety of the Oral Thrombin Inhibitor Dabigatran Etexilate Versus Acetylsalicylic Acid in Patients with Embolic Stroke of Undetermined Source), stroke severity was similarly low (Stroke Scale score 1) [72].

This again points to the dilemma of AF detection in ESUS patients: It is still unknown whether AF represents the cause of the index stroke, whether AF is just incidental, and whether or how AF contributes to stroke recurrence. Unquestionable, prolonged ECG monitoring leads to increased AF detection, regardless of stroke etiology. This was favorably demonstrated in different patient populations: CRYSTAL-AF showed a 12.4% AF-detection rate in cryptogenic stroke [8], which was comparable to 12% in STROKE-AF (Stroke of Known Cause and Underlying Atrial Fibrillation) [41] and 15% in PER DIEM (Post-Embolic Rhythm Detection with Implantable vs. External Monitoring) [42]. Whether AF found in these trials was an asymptomatic background rhythm or an incidental finding due to expanded monitoring is still a matter of debate. This leads to the next question: Do we have to treat incidental bystanders with lifelong anticoagulation therapy? For instance, in NAVIGATE ESUS, >50% of recurrent strokes were atherosclerotic or lacunar [64]. In ASSERT (Asymptomatic Atrial Fibrillation and Stroke Evaluation in Pacemaker Patients and the Atrial Fibrillation Reduction Atrial Pacing Trial) [73], patients with asymptomatic AF lasting >24 h were at higher risk for any cerebral event compared to those with AF lasting <24 h. The latter had the same low risk as those without AF. The temporal association between AF and the risk of ischemic stroke was demonstrated in a recent study: The first five days after a multi-hour episode of AF seemed to be most relevant [60]. There is still a lack of randomized-trial data on the benefit of anticoagulation for asymptomatic-AF patients and the AF burden that should be treated with anticoagulation. Some discouraging results are available from the LOOP study (Atrial Fibrillation Detected by Continuous ECG Monitoring Using Implantable Loop Recorder to Prevent Stroke in High-Risk Individuals) [74]. Irrespective of the three-fold increase in AF detection resulting in anticoagulation treatment, risk of re-stroke did not decrease. Current evidence indicates that in CS, when unknown AF is found, it may be causal in about 38% of patients [75]. Therefore, whether AF was an asymptomatic background rhythm or an incidental finding is still questionable. Following this, in ESUS patients, ILRs may not be inserted routinely but rather on an individualized basis based on specific risk factors such as age, left-atrial size, supraventricular extra beats, and ECG features [76,77,78]. Besides, ECG-monitoring quantification of left-atrial cardiomyopathy has been given great importance because it may double recurrent-stroke risk [79].

### 3.6. Future Directions and Practical Advice

The effectiveness of MCOT depends on several factors, including the patient’s medical history and the reason for monitoring. Different studies have demonstrated that MCOT increases the detection rate of AF by 10 times compared to Holter monitoring. However, it is important to note that MCOT may not be effective for patients with a possible neurological disability after stroke [80]. Handheld ECG-monitoring devices need good compliance [80], even more than the use of chest belts [81]. Placing two fingers from each hand on electrodes for 30 s to record an ECG is not feasible for many post-stroke patients, especially when twice-daily monitoring over a 4-week period is demanded [82,83]. The growing range of MCOT and the differences in usability, sensitivity, and specificity need further assessment in the best case for each model in CS patients. Meanwhile, the value of extended ECG monitoring with ILRs is unquestioned, and gapless ECG monitoring is the best and most effective strategy to significantly increase the detection rate of atrial fibrillation after ischemic stroke. This strategy further supports the use of ILRs without any interruption after the index event as the gold standard in clinical practice. Future developments in artificial-intelligence technologies may facilitate this approach. In summary, an ILR seems to be the best choice for a selected patient group at high risk for atrial fibrillation, most probably leading to a reduced risk of re-stroke and disability.

## Figures and Tables

**Table 1:** Different methods of cardiac monitoring.

Method	Pros	Cons
*Noninvasive*		
**(1) Continuous in-hospital telemetry**	- Accurate diagnosis - Detects asymptomatic events	- Requires inpatient monitoring - Restricts patient movement - Expensive
**(2) Holter-ECG** (24–72 h) **(3) Handheld devices**	- Accurate diagnosis - Detects asymptomatic events	- Short monitoring period - Symptom diary required
Patient triggered/event recorder	- Correlation with symptoms - Longer monitoring periods	- No detection of asymptomatic events - Patient participation required
**(4) Wearables** Mobile automatic/wearable cardiovascular telemetry	- Continuous recording - Detects asymptomatic events	- Patient compliance, skin irritation - Expensive
***Invasive***		
**(5) Implantable loop recorder**	- Follow-up up to 5a - Internet-based data transmission - Detects asymptomatic events - Correlation with symptoms	- False-positive/-negative detection - Initially expensive and invasive
**(6) Already implanted PM or ICD**	- Endless follow-up - Internet-based data transmission - Detects asymptomatic events - Correlation with symptoms	- Restricted to small population group

## Data Availability

Not applicable.
